# Knock-Down of the 37kDa/67kDa Laminin Receptor LRP/LR Impedes Telomerase Activity

**DOI:** 10.1371/journal.pone.0141618

**Published:** 2015-11-06

**Authors:** Kerrilyn Naidoo, Sibusiso T. Malindisa, Tyrone C. Otgaar, Martin Bernert, Bianca Da Costa Dias, Eloise Ferreira, Uwe Reusch, Stefan Knackmuss, Melvyn Little, Stefan F. T. Weiss, Boitelo T. Letsolo

**Affiliations:** 1 School of Molecular and Cell Biology, University of the Witwatersrand, Private Bag 3, Wits, 2050, Johannesburg, Republic of South Africa; 2 Affimed GmbH, Technologiepark, Im Neuenheimer Feld 582, 69120, Heidelberg, Germany; University of South Alabama, UNITED STATES

## Abstract

Cancer has become a major problem worldwide due to its increasing incidence and mortality rates. Both the 37kDa/67kDa laminin receptor (LRP/LR) and telomerase are overexpressed in cancer cells. LRP/LR enhances the invasiveness of cancer cells thereby promoting metastasis, supporting angiogenesis and hampering apoptosis. An essential component of telomerase, hTERT is overexpressed in 85–90% of most cancers. hTERT expression and increased telomerase activity are associated with tumor progression. As LRP/LR and hTERT both play a role in cancer progression, we investigated a possible correlation between LRP/LR and telomerase. LRP/LR and hTERT co-localized in the perinuclear compartment of tumorigenic breast cancer (MDA_MB231) cells and non-tumorigenic human embryonic kidney (HEK293) cells. FLAG^®^ Co-immunoprecipitation assays confirmed an interaction between LRP/LR and hTERT. In addition, flow cytometry revealed that both cell lines displayed high cell surface and intracellular LRP/LR and hTERT levels. Knock-down of LRP/LR by RNAi technology significantly reduced telomerase activity. These results suggest for the first time a novel function of LRP/LR in contributing to telomerase activity. siRNAs targeting LRP/LR may act as a potential alternative therapeutic tool for cancer treatment by (i) blocking metastasis (ii) promoting angiogenesis (iii) inducing apoptosis and (iv) impeding telomerase activity.

## Introduction

Cancer has become a major problem worldwide due to its increasing incidence and mortality rates. According to the World Health Organisation (WHO), cancer accounted for 8.2 million deaths in 2012 alone (http://www.wcrf.org/cancer_statistics/).

The 37kDa/67kDa laminin receptor precursor/ high affinity laminin receptor (LRP/LR) is a high affinity cell surface receptor for laminin-1, an extracellular matrix glycoprotein involved in cell growth, movement, attachment and differentiation (for review: [1, 2]). The relationship between the 67kDa high affinity receptor (LR) and the 37kDa laminin receptor precursor (LRP) remains unknown. LRP/LR is localized on the cell surface as well as in the cytoplasm, perinuclear compartment and the nucleus. The overexpression of LRP/LR is evident in multiple cancer types, and directly correlates with the invasiveness of cancer cells which thereby enhances the risk of cancer metastasis [3–7].

LRP/LR further plays fundamental roles in neurodegenerative disorders such as prion diseases [8–12] and Alzheimer’s Disease [13–17].

Telomeres are specialised DNA-protein structures found at the ends of linear eukaryotic chromosomes. The ends of telomeres have the ability to form a telomere-loop (t-loop) structure [18]. The t-loop is stabilised by the “Shelterin” complex [19]. In this conformation, chromosome ends are protected from degradation and illegitimate processing which could results in premature senescence, recombination and end-to-end fusions and ultimately genome instability; a hallmark of cancer [20–22]. During semi-conservative DNA replication, DNA polymerase fails to replicate the chromosomal ends during the lagging strand synthesis, resulting in the loss of terminal sequences, a phenomenon known as “end replication problem” [23–25]. Cells that are unable to compensate for this mechanism experience progressive telomere shortening, which in turn triggers growth arrest called replicative senescence [26–28]. Replicative senescence is a tumor protective mechanism which cells have to bypass to acquire immortality [29].

Telomeres are maintained and replenished by telomerase. Telomerase is a holoenzyme and a cellular ribonucleoprotein that is involved in the addition of TTAGGG repeats to the 3ʹend of chromosomes. It is composed of two essential components, the enzymatic reverse transcriptase catalytic subunit, hTERT and the integral RNA component, hTR or hTERC [30, 31]. hTERT overexpression and telomerase activity are detected in highly proliferative cells such as embryonic cells, germline cells, adult stem cells and most cancer types [32, 33]. Telomerase stimulates tumor progression by stabilizing the telomeres to prevent the induction of replicative senescence and/or apoptosis. Therefore elevated telomerase activity could prevent a pro-cancer activity and still function as an anti-aging factor by elongating existing telomeres and preventing an accumulation of short telomeres [34, 35].

As LRP/LR and hTERT both play a role in cancer progression and share sub-cellular localizations, we sought to investigate a possible correlation between LRP/LR and telomerase activity.

## Materials and Methods

### Cell culture

Human embryonic kidney cells (HEK293) were cultured in Dulbecco’s Modified Eagle Medium (DMEM) high glucose (Hyclone). MDA_MB231 breast cancer cells were cultured in DMEM/Ham’s-F12 (1:1). All media was supplemented with 10% fetal calf serum (FCS) and 1% penicillin/streptomycin. The cells were cultured at 37°C and 5% CO_2_. Non-tumorigenic HEK293 cells were used as the positive control as they exhibit high telomerase activity whereas the tumorigenic MDA_MB231 cells were used as the experimental model as they are tumorigenic and metastatic.

### Reagents and antibodies

IgG1-iS18 was recombinantly produced in a mammalian expression system as described by Zuber et al., (2008) [36].

### Flow cytometric analysis of cell surface and intracellular levels

Quantification of cell surface and intracellular levels of LRP/LR and hTERT was conducted using flow cytometry. Trypsin/EDTA was used to facilitate detachment of adherent cells which was followed by centrifugation at 1200 rpm for 10 minutes. Cells were subsequently fixed by re-suspending them for 10 minutes at 4°C in 4% paraformaldehyde. Cells were then permeabilised by resuspension in methanol for 30 minutes to detect intracellular levels. Cells were again centrifuged in FACS buffer which allowed for the preparation of two cell suspensions, one to which anti-LRP/LR specific antibody IgG1-iS18 was added to detect LRP/LR and anti-telomerase reverse transcriptase was added to detect hTERT in another. The cell suspension containing only PBS but no antibody was used as the negative control. All suspensions were incubated at room temperature for 1 hour. Following three washing steps with 1X PBS, anti-human-FITC coupled secondary (Sigma Aldrich) was added to each cell suspension to detect LRP/LR and APC-coupled to detect hTERT, followed by another 1 hour incubation period. Furthermore, three post-incubation washes were performed and cell suspensions were analysed using the BD Accuri C6 flow cytometer. The experiments were performed in triplicate and repeated at least three times.

### Confocal microscopy

In order to visualize the co-localization of LRP/LR and hTERT on the cell surface and intracellularly, confocal microscopy was employed. Cells were first seeded on coverslips and allowed to reach 70% confluency. Cells were fixed in 4% formaldehyde in PBS for approximately 15 minutes followed by several washes with PBS. Cells were permeabilised for intracellular visualization with Triton-X BSA solution for 15 minutes followed by several washes. Cells were blocked in 0.5% BSA in PBS for 5–10 minutes. After washing with 1 x PBS, excess PBS was blotted off. The cover slips containing cells were placed on a glass slide (with cells facing upwards) and this was followed by addition of primary antibody IgG1-iS18 (1:100) diluted in 0.5% BSA and anti-Telomerase reverse transcriptase (1:100) diluted in 0.5% BSA and incubated at 4°C overnight. At the end of incubation period, the coverslips were rinsed thrice in PBS/BSA and incubated with FITC-coupled and APC-coupled secondary antibodies (diluted in 0.5% BSA) for 1 hour in the dark. After which, the cells were again rinsed thrice as before. Thereafter, Hoechst 33342 diluted in PBS was administered for 5–10 minutes. Cells were finally washed once in PBS alone and mounted onto a clean slide using GelMount (Sigma-Aldrich). A period of 45 minutes was allocated to allow for setting to take place. Images were acquired at room temperature with 60X magnification using the Olympus IX71 Immunofluorescence Microscope and Olympus XM10 greyscale camera analysis. Research Image Processing Software was used to capture the images.

### FLAG^®^ Co-immunoprecipitation assay (Pull down Assay)

To assess whether there is an interaction between LRP/LR and hTERT, HEK293 cells were transfected with pCIneo-moLRP::FLAG [37] using Lipofectamine 3000 and cultured to stably express the LRP::FLAG as per the manufacturer’s instructions (Invitrogen). The cell lysate was produced from both non-transfected HEK293 cells (lacking the LRP::FLAG) as well as HEK293 cells transfected with pCIneo-moLRP::FLAG. A modified procedure using FLAG^®^ Immunoprecipitation Kit (Sigma-Aldrich) was then used to selectively bind the FLAG peptide. This involved incubating cell lysates with the Anti-FLAG M2 beads in Eppendorf tubes at 4°C overnight. Thereafter, three washes were performed with 1x wash buffer provided in the kit. The washes were then collected as they contained unbound protein. Proteins were then collected and analysed by western blotting. The murine anti-FLAG (Sigma F-3165) (1:4000) and rabbit anti-hTERT (Abcam ab 183105) (1:1000) primary antibodies were used to detect LRP::FLAG and hTERT, respectively. These were then detected using anti-rabbit IgG (Cell signalling 7074S) and anti-murine IgG (Sigma A4416) secondary antibodies coupled with an HRP enzyme.

### Western blotting and SDS-PAGE

Western blotting was used to determine the protein levels of LRP post-transfection with siRNA-LAMR1 when compared to untreated controls, with β-actin used as the loading control. Briefly, cells were lysed, protein levels quantified and 5μg of cell lysate was resolved on a polyacrylamide gel. The proteins were subsequently transferred to a polyvinylidene fluoride (PVDF) membrane for 45 minutes at 350 mV and a semi-dry transferring apparatus. The membrane was blocked in a 1:10 000 solution of the LRP-specific primary antibody IgG1-iS18 in 3% BSA in PBS-Tween at 4°C overnight, with shaking. The membrane was subsequently washed in PBS-Tween, and further incubated in a 1:10 000 solution of anti-human HRP secondary antibody in 3% BSA in PBS-Tween for 1 hour at room temperature with shaking, and washed as before prior to being analysed.

### siRNA-mediated down-regulation of LRP

HEK293 and MDA_MB231 cells were transfected for LRP knockdown with siRNA purchased from Dharmacon, Cat # J-013303-08, according to manufacturer’s instructions using DharmaFECT^®^ 1-transfection reagent. Control siRNA used—Cat # D-001810-04-20. Mission siRNA universal negative control (SIC001 –Sigma Aldrich) was used according to manufacturer’s instructions as a negative control for the experiment.

### Detection of telomerase activity

We utilized the TRAPeze RT^®^ Telomerase detection Kit (Merck Millipore) to determine telomerase activity according to manufacturer’s instructions with minor modifications. Briefly, cells were harvested and washed in PBS. The cells were then lysed with CHAPS lysis buffer. Protein and RNA were collected in the supernatant. Protein concentration was standardized to 500 ng/μl for all experimental and control reactions. All samples were then subjected to experimental analysis accompanied by two controls; heat treatment at 85°C for 10 minutes and control reaction containing a PCR inhibitor as per the manufacturer’s instruction. All reactions were performed in triplicate. The reactions were carried out in the LightCycler LC480 (Roche) under the following cycling conditions: 37°C for 30 minutes, 95°C for 2 minutes and 45 cycles of 95°C for 15 seconds, 59°C for 60 seconds and 45°C for 10 seconds. Telomerase activity was estimated by extrapolation from the standard curve generated by 1:10 serial dilutions (40–0.4 amoles) of TSR8 control as per Merck Millipore instructions. The data was analysed in LightCycler^®^ Software version 1.5.1.

### Data analysis and statistics

Statistical analysis was conducted in Graphpad Prism version 5.03. All statistical analyses were performed using a two-tailed Student’s *t*-test with a 95% confidence interval. *P*-values of less than 0.05 were considered significant. The linear dependencies between two variables were expressed using Pearson’s (r) correlation co-efficient.

## Results

### Human embryonic kidney and metastatic breast cancer cells display LRP/LR and hTERT on the cell surface and intracellularly

**Fig 1:**
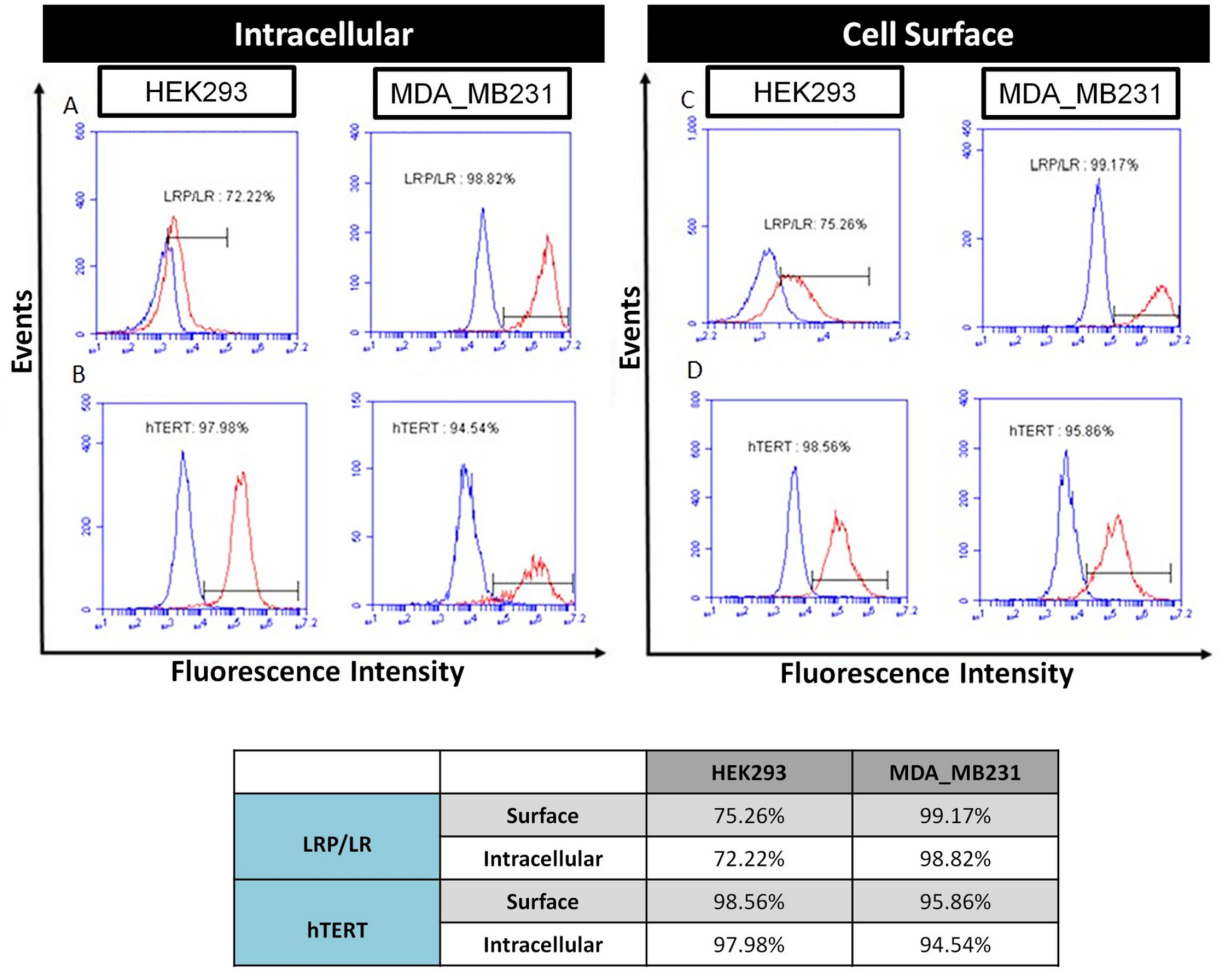
Flow cytometric detection of intracellular and cell surface levels of LRP/LR and hTERT on HEK293 and MDA_MB231 cells. A) Intracellular levels of LRP/LR in permeabilised HEK293 and MDA_MB231 cells were determined primarily by incubating the cells with IgG1-iS18 followed by incubation with anti-human-FITC coupled secondary antibodies (Sigma-Aldrich). B) Intracellular levels of hTERT in permeabilised HEK293 and MDA_MB231 cells were determined primarily by incubating the cells with anti-Telomerase reverse transcriptase antibody followed by incubation with goat anti-mouse IgG-APC coupled secondary antibodies (Sigma-Aldrich). C) Cell surface levels of LRP/LR in non-permeabilised HEK293 and MDA_MB231 cells were determined primarily by incubating the cells with IgG1-iS18 followed by incubation with anti-human-FITC coupled secondary antibodies (Sigma-Aldrich). D) Cell surface levels of hTERT in non-permeabilised HEK293 and MDA_MB231 cells were determined primarily by incubating the cells with anti-telomerase reverse transcriptase antibody followed by incubation with Goat anti-mouse IgG-APC coupled secondary antibodies (Sigma-Aldrich). The blue curve represents the no primary antibody control (to account for non-specificity of the secondary antibodies), whilst the red curve represents cells that were treated with both primary and secondary antibodies. The percentage represents the proportion of cells within the population which expressed LRP/LR and hTERT intracellularly and on the cell surface. This was calculated using a linked marker from the point of intersection between the curves to the end of the red curve.

Since the overexpression of LRP/LR and hTERT has been observed in numerous cancer cell lines, the levels of LRP/LR and hTERT on the cell surface and intracellularly on HEK293 and MDA_MB231 cells were examined by flow cytometry. HEK293 and MDA_MB231 cells displayed high levels of LRP/LR and hTERT on both the cell surface and intracellularly (Fig 1). Of the assessed HEK293 cell population, 72.22% and 97.98% of the cells expressed intracellular LRP/LR and hTERT, respectively. MDA_MB231 cells exhibited 98.82% and 94.54% of LRP/LR and hTERT intracellularly, respectively (Fig 1). The levels of LRP/LR and hTERT on the cell surface were similarly determined. Flow cytometry revealed that 75.26% and 98.56% of HEK293 cells expressed LRP/LR and hTERT on the cell surface, respectively. MDA_MB231 cells expressed 99.17% and 95.86% of LRP/LR and hTERT on the cell surface, respectively (Fig 1). We confirmed previous studies that HEK293 and MDA_MB231 cells display high levels of LRP/LR. However, this is the first report noting the levels of hTERT both intracellularly and on the cell surface on these two cell lines.

### LRP/LR co-localizes with hTERT on the cell surface and in the perinuclear compartments

**Fig 2:**
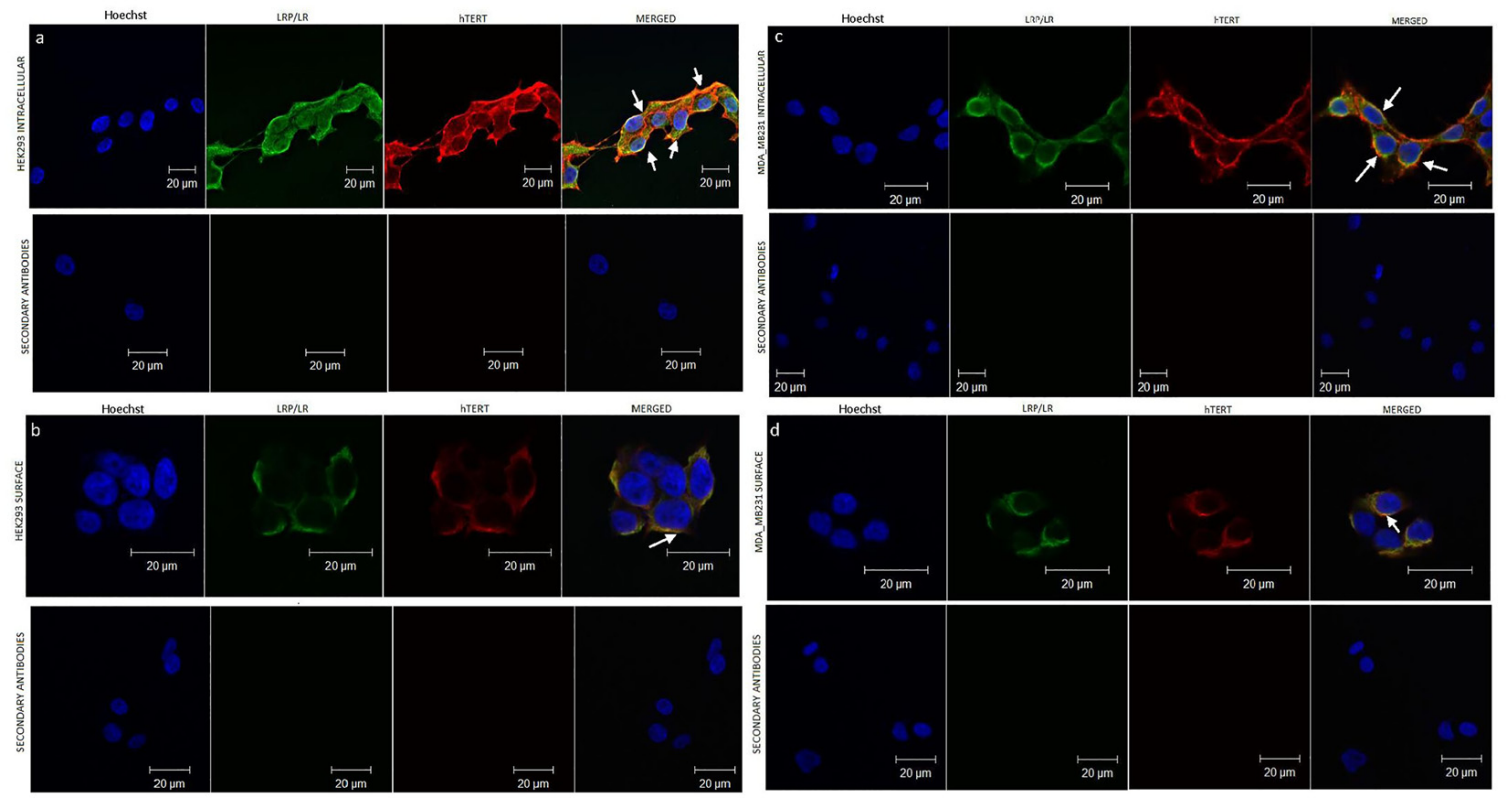
Confocal microscopy analysis of the interaction between LRP/LR and hTERT on MDA_MB231 and HEK293 cells. A) Intracellular LRP/LR and hTERT on immunolabelled HEK293 cells. (B) Endogenous cell surface LRP/LR and hTERT on immunolabelled HEK293. (C) Intracellular LRP/LR and hTERT on immunolabelled MDA_MB231 cells. (D) Endogenous cell surface LRP/LR and hTERT on immunolabelled MDA_MB231. hTERT was detected using anti-telomerase reverse transcriptase and anti-goat to mouse-APC antibodies. LRP/LR was detected employing anti-IgG-iS18 and anti-human-FITC antibodies. Merged images verified the co-localization. The yellow staining indicates areas of co-localization. Secondary antibody controls are shown beneath each panel. Fluorescence was detected and images were acquired using the Olympus IX71 Immunofluorescence Microscope and Analysis Get It Research Software. Scale bars are 20μm. White arrows point to areas of co-localization.

Confocal microscopy was employed to investigate whether LRP/LR and hTERT co-localize on the cell surface as well as intracellularly, as sharing a similar cellular localization may be indicative of a possible association between these proteins. Co-localization of LRP/LR and hTERT was detected on the cell surface of non-permeabilised HEK293 and MDA_MB231 cells (Fig 2b and 2d) and pronounced in the perinuclear compartments of permeabilised HEK293 and MDA_MB231 cells (Fig 2a and 2c). These results demonstrate that LRP/LR and hTERT co-localize in perinuclear compartments and the cell surface but undetectable/no co-localization in the nucleus of the HEK293 and MDA_MB231 cells, respectively.

### FLAG^®^ co-immunprecipitation assay of LRP/LR and hTERT confirms interaction

**Fig 3:**
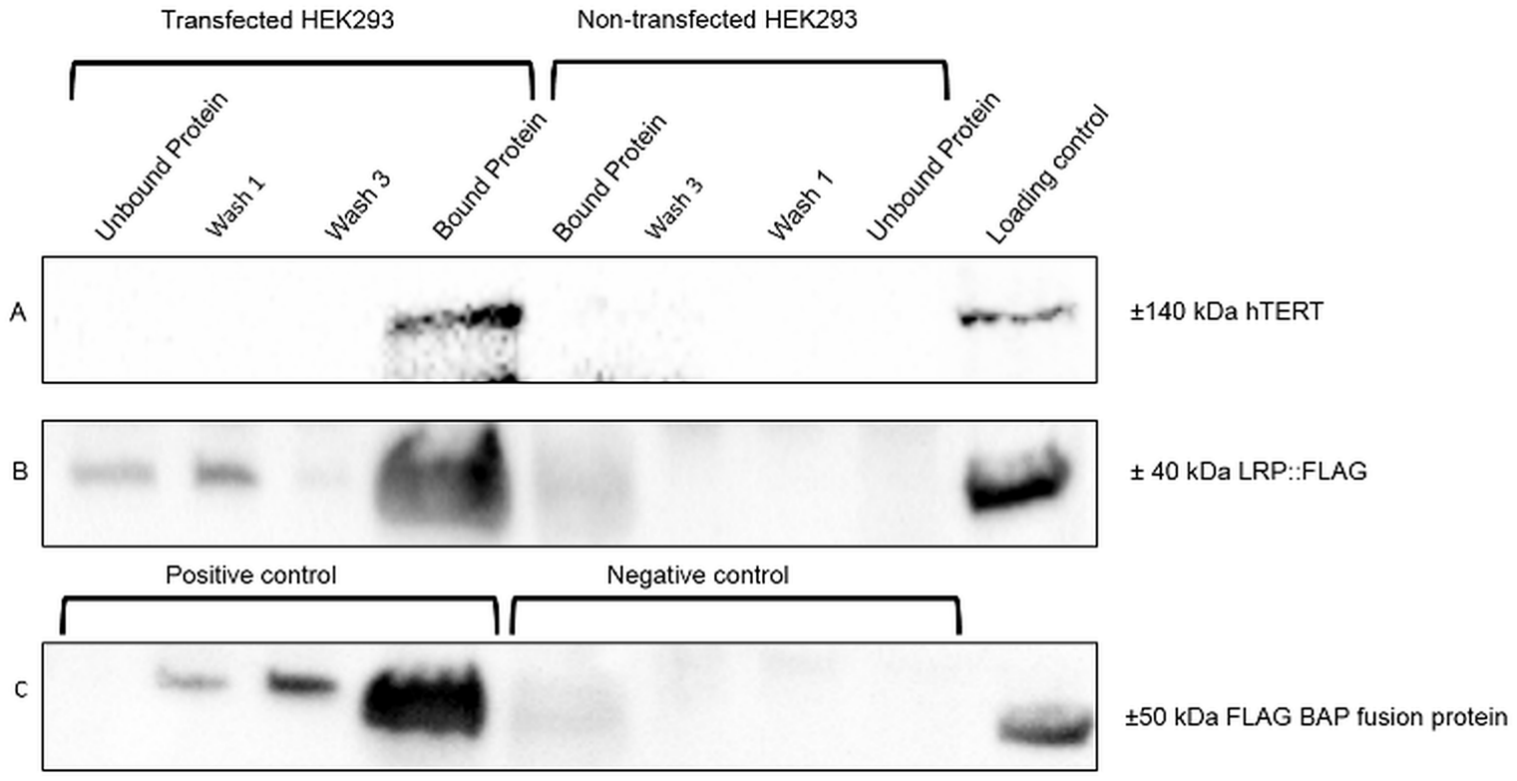
Flag^®^ Immunoprecipitation assays confirming an interaction between LRP/LR and hTERT. Pull down assays were used to detect LRP::FLAG as well as any associated proteins bound to the anti-M2 flag beads. A loading control of crude HEK293 lysate was incorporated to ensure the validity of the blots. Panel C indicates the positive and negative controls, where the Bound protein shows the detection of the BAP fusion protein (50 kDa) to the anti-FLAG beads. Panel B indicates that the LRP::FLAG protein was only present in the HEK293 transfected samples, where FLAG was detected on the anti-FLAG beads (Bound protein). Panel A illustrates the detection of a ±140 kDa band (Bound protein) showing a pull down of hTERT for the HEK293 transfected cell line, whereas no signal was detected for the non-transfected HEK293 cell line.

To assess whether the observed co-localization of the two proteins indicated interaction/association with each other, FLAG^®^ co-immunprecipitation/pull down assays were performed (Fig 3). The presence of the LRP::FLAG and hTERT proteins was detected by corresponding antibodies. h-TERT (panel A, bound protein) and LRP::FLAG (panel B, bound protein) both bound to FLAG^®^M2- beads, in pCIneo-moLRP::FLAG transfected cells, whereas both proteins failed to bind to FLAG^®^-M2 beads in non-transfected cells (bound protein panel A and B, non-transfected cells). This strongly indicates an interaction/association between hTERT and LRP::FLAG.

### siRNA-mediated knockdown of LRP/LR expression in HEK293 and MDA_MB231 cells

**Fig 4:**
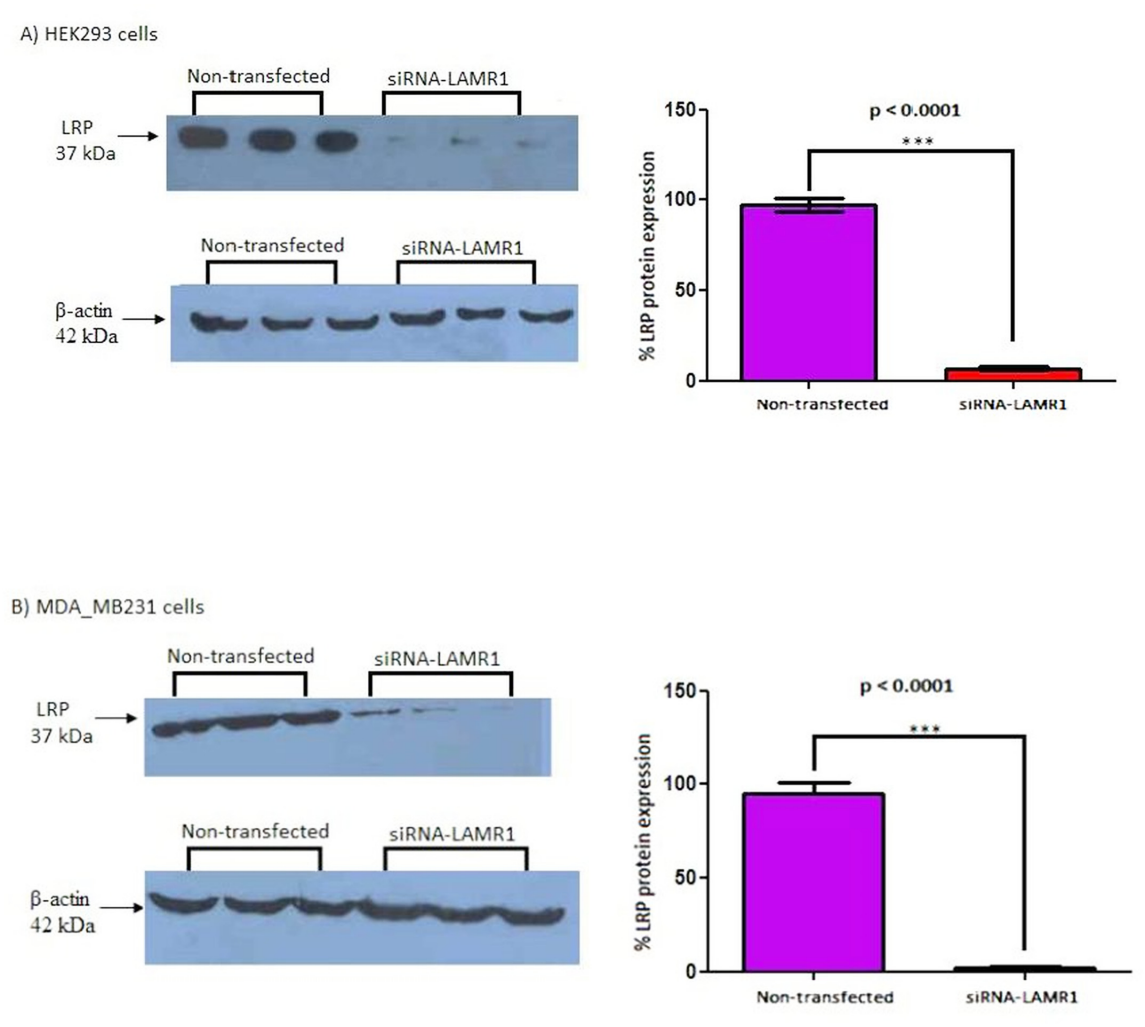
siRNA-mediated knock-down of LRP/LR in HEK293 and MDA_MB231 cells. The expression level in HEK293 and MDA_MB231 cells was investigated 72h post-transfection with siRNA-LAMR1. Densitometric analysis of western blot signals revealed a significant (*** p < 0.001) 90.48% and 92.59% reduction in LRP protein expression in A) HEK293 and B) MDA_MB231 cells, respectively, compared to control non-transfected cells (set at 100%).

To assess whether LRP/LR influences telomerase activity, LRP/LR was down-regulated by employing RNA interference technology using small interfering RNAs (siRNAs). The level of LRP/LR expression in HEK293 and MDA_MB231 cells after transfection with siRNA-LAMR1 was determined using western blotting and was quantified by densitometry (Fig 4). The level of LRP was reduced by 90.48% and 92.59% in HEK293 and MDA_MB231 cells respectively, compared to the non-transfected controls.

### siRNA-mediated knock-down of LRP/LR in HEK293 and MDA_MB231 cells significantly impedes telomerase activity

**Fig 5:**
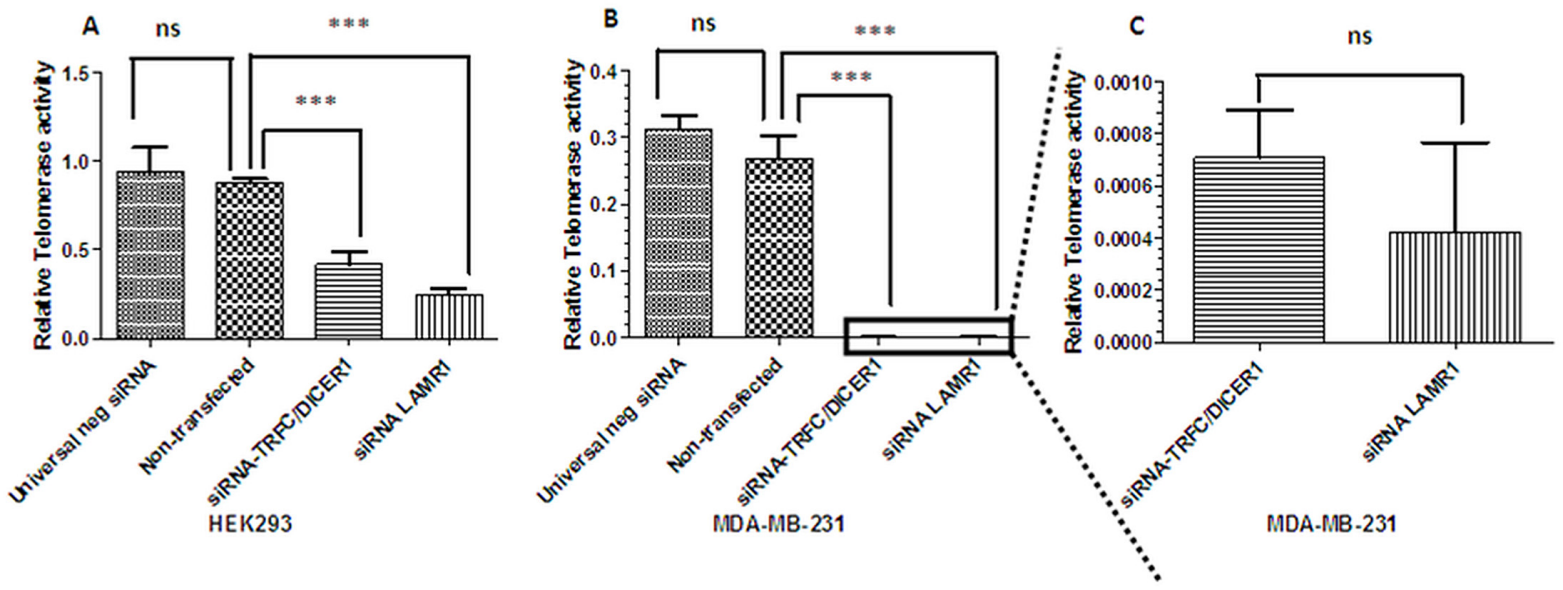
Effect of LRP/LR on telomerase activity in HEK293 and MDA_MB231 cells. The expression level in HEK293 and MDA_MB231 cells was investigated using TRAPEZE Telomerase kit (Merck Millipore) and qPCR. Analysis of the concentrations revealed a significant (*** p < 0.001) reduction in telomerase activity once LRP was knockdown in A) HEK293, B) and C) MDA_MB231 cells, respectively, compared to control non-transfected cells and negative control siRNA transfected cells. Non-significant (ns) at p>0.05.

The telomerase activity in response to the siRNA-mediated down regulation of LRP expression in HEK293 and MDA_MB231 cells was assessed using a TRAPeze RT^®^ telomerase detection kit (Merck Millipore). HEK293 and MDA_MB231 cells were transfected with siRNA-LAMR1 and siRNA-TFRC/DICER1 (positive control). The level of telomerase activity in HEK293 and MDA_MB231 cells was significantly reduced (Fig 5) after the knock-down of LRP/LR.

## Discussion

Numerous studies have implicated LRP/LR and telomerase in the progression of cancer. LRP/LR is overexpressed on the cell surface and intracellularly on a number of cancer cell lines. In these cells, LRP/LR has been shown to confer the ability to metastasize [3–7], hamper apoptosis [38] and promote the induction of tumor angiogenesis [39]. Moreover, telomerase up-regulation enables highly proliferating cancer cells to bypass checkpoint signals during critical telomere shortening which typically induce cellular senescence and cell death (Shay & Wright, 2002). Given that LRP/LR and telomerase are involved in a number of cellular processes and are found in numerous cellular locations (cell surface, nucleus and the perinuclear compartments), an association between these macromolecules warranted investigation.

The tumorigenic breast cancer (MDA_MB231) and human embryonic kidneys (HEK293) cells displayed LRP/LR and hTERT on their cell surface and intracellularly. Flow cytometric analysis revealed that MDA_MB231 cells display higher intracellular levels of LRP/LR and hTERT in comparison to the HEK293 cells. Similarly, the cell surface levels of LRP/LR were higher in the tumorigenic cell line. However, there was no significant difference in the cell surface levels of hTERT between the two cell lines. This is most likely due to the fact that MDA_MB231 cells are tumorigenic and need more LRP/LR and hTERT to maintain their tumorigenic character.

We confirmed previous studies that LRP/LR [3–7] and hTERT are found on the cell surface [40]. The considerably high levels of hTERT on the cell surface may be explained by the presence of an associated peptide known as MHC class1 which is derived from hTERT and is expressed on the cell surface [40]. This peptide may be recognized by hTERT antibodies. These findings demonstrate that LRP/LR and hTERT are found on both the cell surface and intracellularly of both, HEK293 and MDA_MB231 cells.

Confocal microscopy was employed to investigate whether LRP/LR and hTERT co-localize which raises the potential that these proteins may form an association/interaction (Fig 2). The co-localization observed between LRP/LR and hTERT on both HEK293 and MDA_MB231 cells indicates that a spatial overlap occurs between the fluorescent immuno-labelled proteins. Although the close cellular proximity of these proteins on the cell surface of both cell lines has been detected, it does still suggest an association between LRP/LR and hTERT. Confocal microscopy was further utilized to examine whether LRP/LR co-localized with hTERT in sub-cellular locations other than the cell surface. From Fig 2, it is evident that LRP/LR also shows a high degree of co-localization with hTERT within the perinuclear compartment. LRP/LR and hTERT are known to be present in the cytosol, nucleus and perinuclear compartments. From our results, the intracellular distribution of hTERT and LRP/LR is seen to be fairly widespread throughout the perinuclear compartments. This suggests that LRP/LR could be interacting with hTERT within the perinuclear compartments and on the cell surface.

FLAG^®^-Co-immunoprecipitation/pull down assays confirmed that LRP/LR and hTERT interact with each other. This interaction can either be direct or indirect. An indirect interaction could be mediated by proteins present in the crude lysate of the HEK293 cells.

To investigate whether LRP/LR has an effect on telomerase activity, LRP/LR expression was significantly decreased in MDA_MB231 and HEK293 cells by employing RNAi methodology. siRNAs directed against LRP were transfected into the aforementioned cells and the degree of LRP down regulation was assessed by western blotting followed by densitometric analysis (Fig 3). The level of LRP expression was significantly reduced by 90.48% and 92.59% in HEK293 and MDA_MB231 cells, respectively. The effect of the knockdown of LRP expression on telomerase activity was investigated by employing the TRAPeze RT^®^ telomerase detection kit (Merck Millipore) and real-time PCR. LRP down regulation resulted in a significant reduction in telomerase activity in HEK293 and MDA_MB231 cells, respectively, suggesting a crucial role of LRP/LR in telomerase activity. These findings therefore suggest that LRP/LR may play a role in maintaining or enhancing telomerase activity.

Similarly, we observed a significant reduction in telomerase activity after transfection with the siRNA-TFRC/DICER1 positive control. According to Baumer et al., 2010, TFRC and DICER1 enhance telomerase activity [41], which clarifies why the down-regulation of TFRC/DICER1 resulted in a significant decrease of telomerase activity.

The functions of LRP/LR and hTERT in cancer progression are numerous. Targeting LRP/LR can therefore be developed as a strategy to hamper cancer progression. Additionally targeting telomerase activity by down regulating LRP/LR could potentially be an alternative target for cancer therapy as high telomerase expression in breast cancer has been associated with increased death risk, disease recurrence and plausible (possible) resistance to chemotherapy [42, 43]. Thus, targeting LRP/LR via RNAi methodology may serve as an alternative method to target telomerase activity and may thus be beneficial as a two-pronged approach to cancer treatment. i.e targeting LRP/LR could also be used in synergy with other cancer drugs to significantly reduce cancer progression.

The fact that the knock-down of LRP resulted in a significant decrease of hTERT activity, suggests that LRP/LR itself increases telomerase activity. This suggests that LRP/LR or fragments thereof which are responsible for hTERT activation may be used as an anti-aging drug since elongation of telomeres is crucial for implementing (impeding) the aging process. Since low-level telomerase activity can also drive age-related diseases and premature aging syndromes, expression of LRP/LR itself or fragments thereof may be utilized to increase telomerase activity and thus may act as potential therapeutics against these diseases.

In conclusion, we have confirmed that HEK293 and MDA_MB231 cells display hTERT and LRP/LR on the cell surface and intracellularly. The LRP/LR-hTERT interaction, confirmed by FLAG^®^ Co-immunoprecipitation, may occur in the perinuclear compartments and on the cell surface. siRNA mediated knockdown of LRP/LR significantly decreased telomerase activity in HEK293 and MDA_MB231 cells. These findings suggest for the first time a novel function of LRP/LR in contributing to hTERT activity. siRNAs targeting LRP/LR may act as a potential alternative therapeutic tool for cancer treatment by (i) blocking metastasis, (ii) impeding tumor angiogenesis (iii) inducing apoptosis and as demonstrated in this study, by (iv) hampering telomerase activity.
